# Effect of Imagination

**Published:** 1893-12

**Authors:** Frank W. Sage

**Affiliations:** Cincinnati, Ohio


### Domestic Correspondence.

### EFFECT OF IMAGINATION.

Cincinnati, Ohio, August 5, 1893.

To the Editor:

Sir,—I was much interested in reading Dr. Edward Page's article on "Amalgam," in the August number, especially so in the instances he cites showing the effect of imagination on the patient's part, as regards the injurious effect of amalgam fillings. In 1877 I removed from a wisdom-tooth of a lady patient a large amalgam filling, which she declared caused her uneasiness, substituting gold. This she declared relieved her of symptoms of uneasiness of long standing. Nine years later the lingual wall of the tooth broke away, and the filling was lost. By this time I had forgotten about having removed the amalgam filling, and as the lady was in feeble health, unable to endure a protracted operation, I suggested amalgam. This was inserted accordingly, and was in position last week when she called for other services. Meanwhile, in casually looking through my record of operations for 1877, I discovered my notes on this particular case, and inquired whether she recalled the circumstances at that time, and if she had suffered a recurrence of the trouble since the insertion of the second amalgam filling. To both questions she replied in the negative. I should not omit to say that the pulp is still alive.

FRANK W. SAGE, D.D.S.

